# Fronto-Central Changes in Multiple Frequency Bands in Active Tactile Width Discrimination Task

**DOI:** 10.3390/brainsci14090915

**Published:** 2024-09-11

**Authors:** Tiago Ramos, Júlia Ramos, Carla Pais-Vieira, Miguel Pais-Vieira

**Affiliations:** 1 iBiMED—Institute of Biomedicine, Department of Medical Sciences, University of Aveiro, 3810-193 Aveiro, Portugal; tiagoframos@ua.pt (T.R.); julia.ramos@ubi.pt (J.R.); 2 Faculdade de Ciências da Saúde e Enfermagem, Centro de Investigação Interdisciplinar em Saúde (CIIS), Universidade Católica Portuguesa, 4169-005 Porto, Portugal; cvieira@ucp.pt

**Keywords:** active discrimination, EEG, width performance, fronto-central network

## Abstract

The neural basis of tactile processing in humans has been extensively studied; however, the neurophysiological basis of human width discrimination remains relatively unexplored. In particular, the changes that occur in neural networks underlying active tactile width discrimination learning have yet to be described. Here, it is hypothesized that subjects learning to perform the active version of the width discrimination task would present changes in behavioral data and in the neurophysiological activity, specifically in networks of electrodes relevant for tactile and motor processing. The specific hypotheses tested here were that the performance and response latency of subjects would change between the first and the second blocks; the power of the different frequency bands would change between the first and the second blocks; electrode F4 would encode task performance and response latency through changes in the power of the delta, theta, alpha, beta, and low-gamma frequency bands; the relative power in the alpha and beta frequency bands in electrodes C3 and C4 (Interhemispheric Spectral Difference—ISD) would change because of learning between the first and the second blocks. To test this hypothesis, we recorded and analyzed electroencephalographic (EEG) activity while subjects performed a session where they were tested twice (i.e., two different blocks) in an active tactile width discrimination task using their right index finger. Subjects (n = 18) presented high performances (high discrimination accuracy) already in their first block, and therefore no significant improvements were found in the second block. Meanwhile, a reduction in response latency was observed between the two blocks. EEG recordings revealed an increase in power for the low-gamma frequency band (30–45 Hz) for electrodes F3 and C3 from the first to the second block. This change was correlated with neither performance nor latency. Analysis of the neural activity in electrode F4 revealed that the beta frequency band encoded the subjects’ performance. Meanwhile, the delta frequency band in the same electrode revealed a complex pattern where blocks appeared clustered in two different patterns: an Upper Pattern (UP), where power and latency were highly correlated (Rho = 0.950), and a sparser and more uncorrelated Lower Pattern (LP). Blocks belonging to the UP or LP patterns did not differ in performance and were not specific to the first or the second block. However, blocks belonging to the LP presented an increase in response latency, increased variability in performance, and an increased ISD in alpha and beta frequency bands for the pair of electrodes C3–C4, suggesting that the LP may reflect a state related to increased cognitive load or task difficulty. These results suggest that changes in performance and latency in an active tactile width discrimination task are encoded in the delta, alpha, beta, and low-gamma frequency bands in a fronto-central network. The main contribution of this study is therefore related to the description of neural dynamics in frontal and central networks involved in the learning process of active tactile width discrimination.

## 1. Introduction

Tactile discrimination processing in humans and animals is a complex process that involves the integration of multiple types of information, such as sensorimotor and cognitive [1,2,3,4,5]. A small number of studies have recently started to describe the neural correlates of width discrimination in humans [1,4,5], which has been extensively described in rodents [1,4]. As width discrimination in humans plays a key role in tasks such as grasping objects [6], identifying textures [6], and navigating the environment [6], closing this knowledge gap in the human species by exploring neural correlations and somatosensory processing in width discrimination not only enhances our comprehensive understanding of tactile information processing across species but also holds potential for near-future clinical applications [7,8].

In recent years, the development of a width discrimination task [4], as well as other tasks [9], has allowed the study of this function in humans. The width discrimination task can be applied in “Passive” and “Active” versions [5,10]. The Passive version requires a subject to remain with the index finger immobile, while receiving a tactile stimulus, after which the subject must press a button to indicate whether the stimulus delivered was “Wide” or “Narrow”. Meanwhile, the Active version requires the subject to explore the width of the aperture through finger movements. The neural correlates of the passive version of the task have been previously described using EEG recordings [10]. This approach has enabled the characterization of power across relevant frequency bands and electrodes during varying levels of performance in passive tactile width discrimination. Two main networks of electrodes were involved for within-subjects performance (i.e., changes between the first and the second blocks in the same subject) and between-subjects performance encoding (i.e., changes in performance across all subjects and blocks). The between-subjects network encoded the performance of subjects in the task through electrodes spanning Fp2, F4, and P3-4. Meanwhile, the network of electrodes that encoded within-subjects performance, and to some extent their learning and improvement between the first and the second blocks, was associated with electrodes P4 and O2. Namely, it was reported that improvements in performance in a passive version of this task were associated with an increase in information transfer from electrode C3 to electrode F4. These previous results support the notion that for width discrimination, there is a network of electrodes encoding the overall performance of the subject in the task, while another network of electrodes encodes the relative performance, i.e., how much the subject has improved compared to the previous attempt. Such a difference is relevant because it suggests that the networks associated with learning to perform the tactile width discrimination task should differ from the networks associated with keeping track of the overall performance at least for the passive version of the task. Lastly, changes in alpha and beta frequency bands in electrodes C3 and C4, namely through Event-Related Desynchronization (ERD) [11,12] have been previously used to describe the neural processing of motor and tactile stimuli. However, there has been, to date, no description of this type of event in the context of width discrimination. It is expected that learning to perform a width discrimination task should be related with changes in power of the alpha and beta frequency bands associated with these electrodes. In addition, performing the tactile width discrimination task involves not only sampling a width aperture formed by the two movable bars but also preparing and performing the appropriate behavioral response [5,10]. This means that changes in latency resulting from learning also have the potential to change Bereitschafts potentials (BPs), which are changes occurring in low-frequency bands of the EEG signal of electrodes C3–C4 and that reflect motor preparation for a behavioral response [13,14].

Here, we hypothesized that subjects learning to perform the active version of the width discrimination task would present changes in behavioral performance, response latency, and in the neurophysiological activity in networks of electrodes relevant for tactile and motor processing. For this, subjects were tested twice (i.e., two blocks of 20 trials) in the active version of the width discrimination task, while neural activity was recorded using electroencephalography (EEG). Our first hypothesis (H1) was that the performance and response latency of subjects would change between the first and the second blocks [10]. The second hypothesis (H2) was that the power of the different frequency bands would change between the first and the second blocks [10]. The third hypothesis (H3) was that electrode F4 would encode task performance and response latency through changes in the power of the delta, theta, alpha, beta, and low-gamma frequency bands [10]. Lastly, our fourth hypothesis (H4) was that the relative power in the alpha and beta frequency bands in electrodes C3 and C4 (from now on referred to as Interhemispheric Spectral Difference—ISD) would change as a result of learning between the first and the second blocks [11,12].

## 2. Materials and Methods

The present study was approved by the Ethics Committee of the University of Minho (SECVS 148/2016) and the Comité para as Ciências da Saúde of the Catholic University of Portugal (39/2017) in accordance with the Code of Ethics of the World Medical Association (Declaration of Helsinki) for experiments involving humans. All experiments were performed in accordance with relevant named guidelines and regulations. All participating subjects voluntarily filed an informed consent. All subjects were tested at the Catholic University of Portugal.

### 2.1. Subjects

A total of 18 subjects asymptomatic for neurological and sensory motor disorders were initially tested. A brief interview was conducted before testing to ensure that participants were capable of performing the task. The subjects consisted of 8 males (44.4%) and 10 females (55.6%) with an average age (M ± SD) of 31.0 ± 7.24 years (the minimum age was 18 years, and the maximum was 40 years). Three subjects were left handed. Four subjects (S1, S2, S5, and S17) only performed one block with a total of 20 trials. The neural data of one subject (S17) were not analyzed due to technical problems. Another subject (S15) completed both blocks, but the neural data (EEG recordings of power levels from each subject) were 3.2 standard deviations (for electrode Fp2 (frontal cortex)) above the remaining sample and was therefore considered an outlier and was not analyzed. Moreover, the Fp2 electrode did not show any outlier values for any other subject, reinforcing the premise that S15 data for this electrode might be compromised by noise or technical issues [15]. Fourteen subjects completed both blocks (N = 14 sessions, a total of 28 blocks), and the neurophysiological data of sixteen subjects were analyzed (N = 16 sessions, a total of 29 blocks). Due to these experimental design contingencies, the number of blocks and subjects analyzed in each experiment is specified in each subsection.

#### Width Discrimination Task

**Figure 1:**
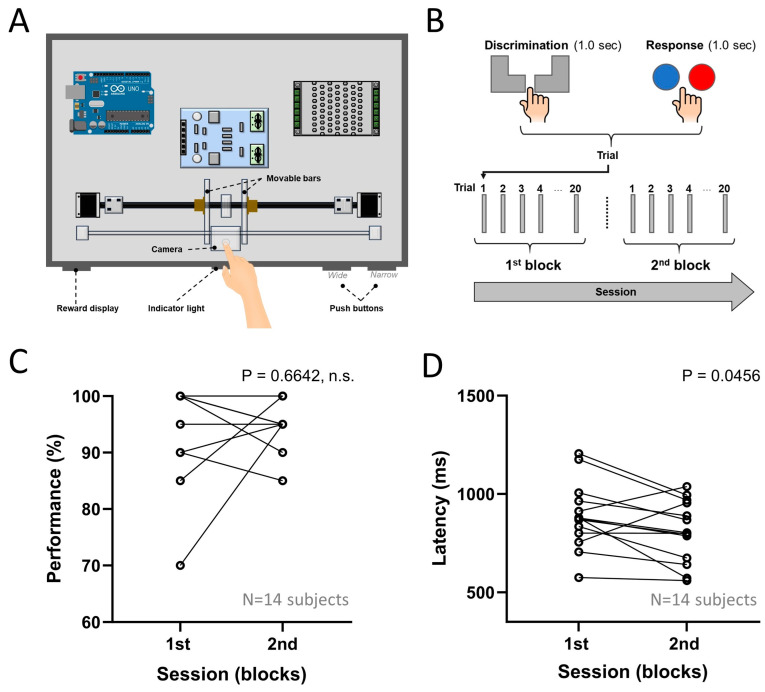
Study design and behavioral performance. (**A**) Tactile width discrimination box depicting the subjects’ finger, movable bars, camera, indicator light, reward display, and push buttons. (**B**) Each trial included a discrimination (i.e., the Wide or Narrow stimulus) and a response period (i.e., pressing one of the push buttons). A block was composed of a set of 20 trials (10 Narrow and 10 Wide). A session was composed of a set of two blocks. Representation of “Wide” and “Narrow” stimulus delivered in the tactile width discrimination task. (**C**) No significant improvement was found between the first and the second blocks. n.s. indicates a non-significant comparison. (**D**) There was a significant reduction in latency between the first and the second blocks. Note that some subjects presented equal performance in the first and/or in the second blocks, and therefore one circle may represent more than one subject.

The width discrimination task has been previously described in detail [5]. Briefly, it consisted of a box with a front panel where the subject inserted the right index finger (Figure 1A). In each trial, a yellow light signaled the subject to insert the finger. A computer vision algorithm, fed by the signal streamed from a camera placed behind the front panel, detected when the finger was in the appropriate position, switching the front panel light to green. Two movable bars then created either a “Narrow” or “Wide” aperture (Figure 1C), providing tactile stimulation as the subject moved their finger through the aperture (Discrimination Period). During this interval, the software automatically generated a timestamp (Discrimination), which was then recorded in a text file for posterior analysis. The stepper motors were controlled by an Arduino Mega 2560, which was chosen for its ease of use and large community support. After the tactile stimulation, the bars reverted to their initial positions, which was followed by a red light signaling the subject to remove the finger and press a pushbutton (indicating the type of stimulus delivered) (Figure 1B). When the subject pressed one of the pushbuttons, a second timestamp was automatically recorded by the software (Figure 1B) (Response). For each subject, the finger width was measured and used to calibrate the movable bars. During the task, the Narrow stimulus was delivered as +0.0 cm on each side of the finger, and the Wide stimulus was delivered with a width of +0.25 cm on each side of the finger (a total increase in width of 0.5 cm). Therefore, the absolute distance between Wide and Narrow varied by subject due to individual finger width.

### 2.2. EEG Recording and Pre-Processing

Electroencephalographic recordings were made from 16 electrode channels at 1000 Hz using a 10–20 placement (V-Amp, actiCAP; Brain Products GmbH, Gilching, Germany). Signals were recorded using the Brain Vision Recorder (version 2.1.0, Brain Products, Gilching, Germany) and analyzed using Brain Vision Analyzer (version 2.2.1, Brain Products, Gilching, Germany) and Matlab (Mathworks, 2023b, Natick, MA, USA). Before the EEG recording began, subjects were sequentially instructed to (i) chew three times, (ii) blink three times, and (iii) close their eyes and remain still and silent for 5 s. The expected changes in the EEG signals were confirmed by the researchers in real time. Pre-processing included re-referencing, using all channels as reference [16]. A Notch filter was applied (50 Hz). A zero-phase shift, 4th-order Butterworth filter with a low cutoff of 0.5 Hz and a high cutoff of 70 Hz with a time constant of 0.3183 was used. Ocular correction was performed using the Gratton and Coles algorithm (already implemented in Visual Analyzer).

#### EEG Power and Processing

The data were segmented based on the discrimination and response markers generated by the tactile discrimination task software, using a window of 1000 ms (−500 to +500 ms around the discrimination marker—Discrimination Period). Subsequently, Fast Fourier Transform (FFT) with a resolution of 0.5 Hz was applied. Power was analyzed across frequency bands: delta (0.5–4.0 Hz), theta (4.5–8.0 Hz), alpha (8.0–13.0 Hz), beta (13.0–30.0 Hz), and low gamma (30.0–45 Hz). Data analysis was limited to 45 Hz (referred to as the low-gamma frequency band here) to align with previous analyses [10,17]. Power values were then normalized for all subjects and are therefore presented as Z scores with arbitrary units (a.u.). It should be noted that Z scores may appear as negative values due to this normalization. During the active discrimination task, the discrimination period was compared between the first and second blocks. Fourteen subjects were analyzed (considering those who completed both trials), totaling N = 28 blocks. The same comparison was then repeated, excluding one subject (S15), which presented extreme values and was considered an outlier. Interhemispheric Spectral Difference (ISD) was used as a measure of sensorimotor processing [11,12] and was calculated as follows: (i) the mean power for the alpha and the beta frequency bands was calculated for electrodes C3 and C4 (somatosensory cortex); and (ii) the difference between the two means was calculated (C3–C4). The comparison of power was made employing the exact permutation test for each subject and electrodes.

The study used N = 29 blocks from 16 subjects to analyze how the power of specific frequency bands correlates with individual performance in the task. Two subjects (S15 and S17) were excluded from this analysis: S17 was excluded due to technical problems, while S15 showed neural data for electrode Fp2 (right pre-frontal lobe) to be 3.2 standard deviations from the data sample even after repeating the comparison. The power values of both sessions were compared to their respective performance and latency values using Spearman’s Rho correlation.

### 2.3. Statistical Analysis

Results are presented as mean and standard deviation (mean ± SD). Data analysis was performed using non-parametric tests due to the non-Gaussian nature of the EEG signal [18]. Exact permutation tests [19] for comparison of power in the different frequency bands were performed using MATLAB software (Mathworks, 2023b, Natick, MA, USA) [20], utilizing a parallel computing package [21]. Spearman’s Rho was used to correlate the power in beta and gamma frequency bands with performance or latency or to compare latency and performance. For all tests with multiple comparisons, the alpha value was corrected using the Benjamini and Hochberg correction for false discovery rate [9]. Paired samples *t*-tests or independent samples *t*-tests were employed to compare the behavioral performance and latency values between blocks as well as variables calculated from these (e.g., changes in performance). When variance was heteroscedastic, the non-parametric alternatives Mann–Whitney test or Wilcoxon rank-sum test were used. Significance was considered at an alpha level of 5%. The previous statistical tests were conducted using the GraphPad Prism software [22] as well as Matlab (Mathworks, 2023b, Natick, MA, USA) [20].

## 3. Results

### 3.1. Behavioral Results

To test our first hypothesis (H1), the performance and latency between the first and the second blocks were compared. As presented in Figure 1D, for the fourteen subjects who completed both blocks, behavioral performance was high already in the first block and, in addition, was not significantly different between the first and the second blocks (first block: 95.0 ± 8.45% correct; second block: 96.1 ± 4.30%; Paired samples *t*-test: t = 0.444; df = 13, *p* = 0.664, n.s.). Meanwhile, as presented in Figure 1D, a significant decrease in response latency was observed, for the same fourteen subjects, between the first and second blocks (first block: 888.05 ± 161.19 milliseconds; second block: 810.21 ± 148.48 milliseconds; paired samples *t*-test: t = 2.21; df = 13, *p* = 0.0456). The changes between the first and the second block in performance were not correlated to the changes in latency (Rho = −0.1667; df = 12; *p* = 0.569, n.s.).

### 3.2. Neurophysiological Analysis

#### Power Changes between Blocks

To test our second hypothesis (H2), the power for the different frequency bands was compared in the first and the second blocks for the thirteen subjects (N = 13). Analysis of power for the different frequency bands (delta (0.5–4.5 Hz), theta (4.5–8.5 Hz), alpha (8.5–13.5 Hz), beta (13.5–30.5 Hz), and low gamma (30.5–45 Hz)) across 16 electrodes between the first and second blocks of the active discrimination task (N = 26 blocks in 13 subjects) revealed increases in low-gamma frequency for electrodes F3 (1st block: −0.0973 ± 0.0404; 2nd block: −0.0815 ± 0.0654; *p* = 0.01601) and C3 (1st block: −0.0983 ± 0.0422; 2nd block: −0.0618 ± 0.0828; *p* = 0.03580). A summary of the power for all frequency bands in all electrodes recorded is presented in Table 1.

**Figure 2:**
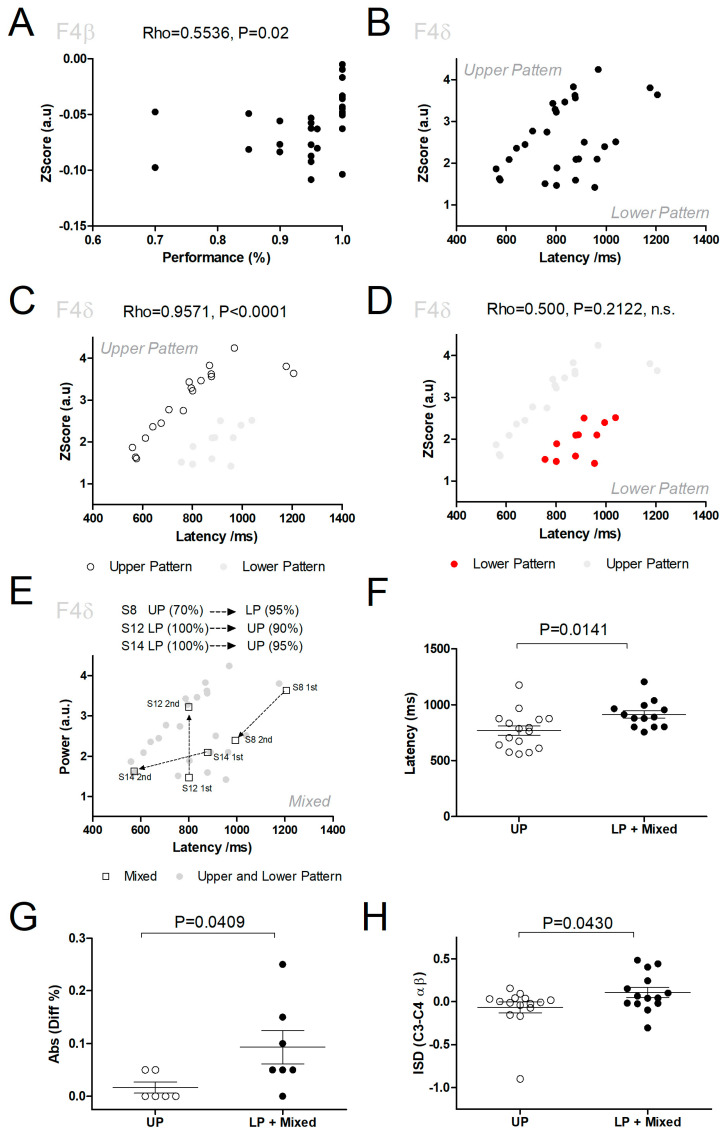
F4 electrode encodes latency and performance in different frequency bands. (**A**) The behavioral performance in the task (both blocks) could be predicted from power in the beta frequency band in the F4 electrode. (**B**) Analysis of latency in the delta frequency band revealed that blocks could be associated with an Upper Pattern (UP) and a Lower Pattern (LP). (**C**) The UP (empty circles) encoded latency with a near perfect correlation. (**D**) The LP (red circles) did not present a significant correlation with latency. n.s. indicates a non-significant correlation. (**E**) A small number of subjects presented a Mixed pattern (S8, S12, S14) (empty squares with arrows starting in the first block and ending in the second block), where one block was associated with the UP and another with the LP. Subject S8 moved from the UP in the first block to LP in the second block, and an increase in task performance was observed. Meanwhile, subjects S12 and S14 moved from LP in the first block to UP in the second block, and a decrease in task performance was observed. (**F**) Subjects in LP and with Mixed patterns presented increased response latencies. (**G**) Subjects in LP and with Mixed patterns presented increased absolute variability in their performance (i.e., presented larger increases as well as decreases). (**H**) Subjects with Mixed patterns and in the LP in electrode F4 presented an increase in ISD in electrodes C3–C4 for the alpha and beta frequency bands, suggesting an increase in sensorimotor processing for LP.

To test our third hypothesis (H3), that neural activity in F4 encoded the task’s behavioral variables, the power in the different frequency bands for this electrode was correlated with the subjects’ performance. As presented in Table 2 and in more detail in Figure 2A, a significant correlation between the beta frequency band and task performance was observed (Rho = 0.554; df = 27, *p* = 0.0200). Meanwhile, analysis of the delta frequency band, which was not statistically significant (Rho = 0.356; df = 27, *p* = 0.247), revealed a pattern suggestive of two fundamentally different brain states, as presented in Figure 2B. More specifically, a part of the sample indicated an almost linear pattern between latency and power in the delta frequency band (Upper Pattern), while another indicated a non-linear pattern, which was associated with a clustering around the 800–1000 ms and power in the 1–3 a.u. range. Detailed analysis of these two patterns—from now on termed the Upper Pattern (UP) and the Lower Pattern (LP)—revealed that some subjects appeared solely on the UP (UP: S1–3, S5, S9–11, S16, and S18) (Figure 2C), some subjects appeared solely in the LP (LP: S4, S6, S7, and S13) Figure 2D, and lastly, three subjects had one block in each pattern (Mixed: S8, S12, and S14) (Figure 2E, empty squares connected by arrows). A comparison of performances revealed no differences (UP: 95.5% ± 0.0800; LP + Mixed: 93.2% ± 0.0900, Mann–Whitney: rank sum = 243.5, *p* = 0.406). Also, no differences were found in performance variation between the two blocks (i.e., Block 2 and Block 1) of subjects in the UP or LP + Mixed revealed (UP: −1.59 × 10^−17^ ± 0.029; LP + Mixed: 0.0214 ± 0.129; *p* = 0.359, n.s.). However, comparison of latencies revealed that these were higher in the LP + Mixed subjects (UP: 780.672 ms ± 164.129; LP + Mixed: 888.786 ± 148.155, Mann–Whitney: rank sum = 175, *p* = 0.0307) (Figure 2F) and the size of the changes in performance (i.e., absolute value of the difference between the performance in the first and the second blocks) was significantly different between subjects in UP and subjects in LP + Mixed (UP: 0.0143 ± 0.0244; LP + Mixed: 0.0929 ± 0.0838; *p* = 0.0207) (Figure 2G). Thus, subjects that had blocks with the F4 theta frequency band in the LP (i.e., LP and Mixed) presented higher variability in their performance and took longer to respond during the task.

Further analysis of the Mixed subjects, that is, subjects that presented one block in the UP and one block in the LP (S8, S12, and S14), revealed that moving to the LP was associated with an increase in performance (S8), while moving from the LP to the UP was associated with a decrease in performance (S12, S14) (Figure 2E). As these results indicated that the UP and LP brain states corresponded to different behavioral states, we then tested if neural activity in the electrodes C3-C4, which is a hallmark of sensorimotor processing [11,12], was associated with each of these two states. For this, we compared the difference in power between these two electrodes for the alpha and beta frequency bands.

A comparison of the Interhemispheric Spectral Difference (ISD) between subjects that were on the UP (S1–3, S5, S9–11, S15–16, and S18) with the subjects that were on the LP (S4, S6, S7, and S13) or in Mixed (S8, S12, and S14) revealed that subjects in the UP presented lower ISD values (Upper line subjects: −0.00530 ± 0.125; Other subjects: 0.0916 ± 0.1195; *p* = 0.0273). Meanwhile, no significant difference was observed in ISD between the first and the second blocks (1st block: 0.0377 ± 0.0955; 2nd block: 0.0393 ± 0.162; *p* = 0.978), therefore not supporting the fourth hypothesis (H4).

## 4. Discussion

We have compared the behavioral performance and the neurophysiological activity of subjects performing two blocks in an active version of the tactile width discrimination task. No differences between the first and the second blocks were found in the behavioral performance with subjects presenting very high performances already in the first block. Meanwhile, a decrease in latency was found between the first and the second block, suggesting some form of learning or improvement. Analysis of neural activity revealed an increase in power for the second block in electrodes C3 (left central lobe) and F3 (left frontal lobe) for the low-gamma frequency band. The F4 (right frontal lobe) electrode revealed a significant correlation with performance in the beta frequency band, while two different patterns (UP and LP) occurred in the delta frequency band. Subjects in the UP presented an almost perfect correlation between power in the delta frequency band with response latency, while no correlation was found for the LP. Subjects in the LP (in the F4 electrode) also presented an increase in response latency, increased variability in performance between the two blocks, and increased ISD (in electrodes C3–C4), suggesting that the LP state was associated with increased cognitive load in the task. Lastly, subjects that presented one block in the UP and another in LP all presented lower performances in the UP. All together, these results suggest that changes in performance and response latency in an active width discrimination task are dependent on a frontal–central asymmetrical network involving the delta, alpha, beta, and low-gamma frequency bands. This study demonstrates different neural states occurring during active tactile width discrimination learning.

No differences were found in the performance between the first and the second blocks, suggesting that a ceiling effect occurred [16]. Part of this effect may be explained by the fact that some subjects had previous contact with a passive version of the task [10]. Also, the median performance was slightly higher than the one previously reported by us for a small group of subjects tested in the active version of the task [4]. Despite the lack of differences in performance, a reduction in latency occurred between the two blocks, which suggests that some form of improvement or learning occurred. Therefore, these results partially support our hypothesis (H1) that learning to perform the active version of the width discrimination task would be associated with changes in behavioral parameters to the extent that response latency was reduced.

The power analysis in the different frequency bands in the first and the second blocks demonstrated that an increase in power was found for electrodes C3 and F3 in the low-gamma frequency band. This finding supports our second hypothesis (H2). An overall tendency toward an increase in power for the same frequency band in the same electrodes has been previously reported by us for a passive version of the task [10]. The C3 electrode is positioned above the right somatosensory cortex and has been described as being relevant for other tactile tasks [23]. Namely, the gamma frequency band has been reported to be relevant for perceptual processing in the activated cortex, possibly reflecting cognitive functions such as working memory [23], which is likely to be involved with the ability to maintain the estimated width (i.e., Wide or Narrow) while the subject moves the finger toward the pushbutton to make a response in each trial. Here, we have only analyzed the low-gamma frequency band (30–45 Hz), because we have previously observed that it is relevant for the tactile width discrimination task [10].

The F3 electrode also presented an increase in power for the low-gamma frequency band. It has been previously described [24] that a network involving the ipsilateral occipital cortex, the right posterior parietal cortex, and the contralateral prefrontal cortex contributes to tactile discrimination using a feedforward network in the beta frequency band and a recurrent network in the gamma frequency band that did not include the occipital cortex. Additionally, according to a previous study [24], the gamma (80 Hz) network oscillations create a recurring loop from the prefrontal to posterior parietal to somatosensory areas, implying their role in selecting important sensory information. Moreover, there is also an increase specifically in the gamma band activity observed in the posterior Intraparietal Sulcus (pIPS) and the dorsal Inferior Prefrontal Cortex (dIPFC). So, this increase in low-gamma frequency power at electrode F3 supports its involvement in modulating attentional mechanisms related with sensory details. Our F3 findings are partially aligned with these results to the extent that changes in the F3 and C3 electrodes were present throughout the interval analyzed here. It should be noticed, however, that the intervals analyzed by Adhikari and colleagues [24] were much smaller than the ones analyzed here.

The power of the beta frequency band in electrode F4 was predictive of the task performance. This finding supports our third hypothesis (H3) and partially supports our previous findings [10] where a ratio including the beta and low-gamma frequency bands also encoded task performance in a passive version of this task. It is unclear, at this point, if the correlations found between frontal electrodes and performance in this, and the previous study [10], are associated with a prefrontal gating or attentional mechanism [25,26]. The analysis of latency in the F4 electrode revealed two different patterns (UP and LP) suggesting that the delta frequency band encodes two different brain states related to task difficulty and/or attention. Namely, the power in the delta frequency band revealed a complex pattern where on one side, the UP was almost perfectly associated with the response latency, while the LP was not. In addition, the LP presented an overall increase in Event-Related Desynchronization in electrodes C3–C4 (somatosensory cortex) as well as a larger variability in their performance. In other words, subjects that presented one or both blocks in the Lower Pattern (i.e., LP and Mixed) tended to take longer to make a response, and their performance varied more. Moreover, all subjects from the Mixed group (S8, S12, and S14) presented lower performances in blocks that fitted the UP. These findings seem to support the existence of two different states—LP and UP—where the LP occurs when subjects take a long time to make a response, their performance changes, and an increased ISD is present. Such a network, involving F4–C3–C4 (right frontal lobe and somatosensory network) seems to be partially in line with studies from our and other groups in regard to learning, attention, and tactile processing [10,26]. It remains, however, to be determined to which extent the network here reported reflects a change in tactile information (e.g., improved decoding), a change in motor performance (e.g., different motor strategy) or both. Dissecting the mechanism underlying these different components will be the subject of future studies. Lastly, it should be noted that even though we have identified a relevant relation between ISD in C3–C4 and F4, our fourth hypothesis (H4), that the ISD would change between the first and second blocks, was not supported by the present results. A potential explanation for this is the very high performance of subjects in the first block.

We have reported here that one network of electrodes involving the left frontal (F3) and the left central (C3) electrodes changed power in the low-gamma frequency band as subjects performed two active blocks of the width discrimination task and reduced their response latency. We have also described another network where the right frontal (F4) electrode encoded performance and latency in two different frequency bands (beta and delta, respectively) and potentially interacted with the C3–C4 electrodes through the alpha and beta frequency bands. In a previous study conducted in the passive version of this task, we reported that a network of electrodes involving the fronto-parietal cortex was correlated to the between-subjects task performance and that within-subjects changes [10] (i.e., between the first and second blocks of the passive task) were associated with a network of electrodes involving the parietal–occipital regions (P4–O2) in the lower-frequency bands (delta and theta frequency bands). These previous findings suggested the presence of two different networks of electrodes, where one was associated with the actual performance while the other was associated with the relative performance (possibly reflecting attention and/or cognitive load). Comparison of the first and second blocks for electrodes P4 (right parietal lobe) and O2 (right occipital lobe) did not reveal changes for either electrode, suggesting that the active version of the task may be associated with a different network of electrodes, or otherwise, the very high performance already observed in the first block did not allow detecting differences in neurophysiological activity for this specific group of electrodes. Meanwhile, the present description of a fronto-central network with distinct dynamics that seem to be associated with the cognitive load suggests that if there is a network associated with keeping track of the relative performance, it should probably include electrodes F4 as well as C3 and C4 (right central lobe)(Table 3). Future studies will allow studying these interactions in more detail.

Up to this point, the discussion has primarily focused on the encoding of tactile stimuli. However, successfully performing the width discrimination task requires not only the ability to differentiate between two widths but also the preparation and execution of the appropriate motor response when pressing a button. The reduction in response latency observed between the first and second blocks may therefore also be attributed to improvements in motor planning and execution.

Bereitschafts potentials (BPs) are EEG changes in low-frequency bands associated with motor preparation [13,14]. BPs consist of an early phase (occurring more than 1000 ms before movement) and a late phase (approximately 500 ms before movement), which are believed to originate from the supplementary motor area and the primary motor cortex [13,14]. Although increased contralateral activity in lower-frequency bands at the C3–C4 electrodes has been linked to BPs, it remains unclear how these changes relate to the UP and LP states observed in this study. On one hand, the changes in C3–C4 activity and the reduction in response latency suggest improvements in motor preparation and execution. On the other hand, BPs are typically associated with changes at the Cz electrode, which were not detected here [14]. In future studies, it will be relevant to distinguish between the effects of tactile stimuli processing and behavioral response preparation through an increase in the interval between tactile stimulus sampling and behavioral response.

As the description of the neural correlates of width discrimination has mostly been based on rodents [1,4], one of the goals of the present study was to improve our current knowledge on the neural basis of width discrimination in humans. The present results are in line with previous results in rodents, supporting the notion that a role for frontal regions/electrodes is critical for sensorimotor integration during width discrimination [4]. In addition, it also raises the possibility of specifically testing the role of delta, alpha, beta, and low-gamma frequency bands in fronto-central circuits in the width discrimination task for rodents [1,4].

A small number of limitations should be considered in the present study.

Firstly, although the initial number of subjects was appropriate, the final sample size analyzed was relatively small. This means that even though we have found significant differences between the first and the second blocks as well as a significant correlation between the activity of electrode F4 and performance, it is possible that other relevant results could have been identified if a larger sample had been studied. In addition, three of the subjects were left handed, which could have influenced, to some extent, the results obtained.

Secondly, due to the technical details of the task, the interval for the analysis of electrophysiological activity was relatively large (i.e., 1000 ms) when compared to other studies [24]. This disparity impedes a direct comparison of electrophysiological findings.

Thirdly, we have calculated ISD as a measure of sensorimotor processing, namely through the difference between C3 and C4 electrodes (for alpha and beta frequency bands) during the discrimination period. We have opted for this measure, instead of ERD [11,12], due to the variable inter-trial interval present in this task, the large variability in motor strategies, and the potential for additional variables to be involved [5,10]. Additionally, we have used both the alpha and beta frequency bands for ISD calculation. It has been previously demonstrated that the beta band is associated with motor ERD, while the alpha band is associated with tactile ERD [11]. As we could not be certain whether subjects presented differences due to the motor strategy (e.g., placing the finger in a specific position), to tactile encoding, or a combination of both, we chose to utilize the mean of the two frequency bands when analyzing the ISD. One other aspect to be considered is that we have restricted our analysis of the gamma frequency band to its lowest portion (30–45 Hz), since we have previously observed that this seems to be the most relevant for the present task [10]. It cannot be excluded, however, that such delimitation may lead to a loss of other results that could appear in the higher-gamma frequency band.

It is important to note that participants were required to press one of two buttons, which could have influenced the results to some degree. However, given the close proximity of the buttons and the relatively long intervals analyzed (1000 ms), any such differences are expected to be minimized in the analysis.

Lastly, our subjects presented very high behavioral performances already in the first block, which led to a ceiling effect [16] and prevented comparisons with neural activity and with our previous study [10].

## 5. Conclusions

The present study described the neural correlates of width discrimination as subjects performed an active version of the task twice and identified neural dynamics in frontal and central networks during the learning process. A frontocentral network involving the low-gamma frequency band was associated with differences between the first and the second block. Task performance and response latency were, respectively, encoded in the beta and delta frequency bands of the electrode F4. Also, the electrode F4 for delta showed two distinct patterns (LP and UP), which were characterized by changes in performance, response time, and Interhemispheric Spectral Difference (ISD) in the fronto-central network. Moreover, the encoding of latency in the F4 electrode was associated with the relative power of the alpha and beta frequency bands in electrodes C3 and C4. While the previous study, conducted using the passive version of the task, identified a network associated with parietal and occipital electrodes that reflected changes between the first and second blocks, the current study, conducted using the active version of the task, identified a network involving fronto-central electrodes. These results support the notion that active tactile width discrimination is dependent on a fronto-central network involving the delta, alpha, beta, and low-gamma frequency bands.

## Figures and Tables

**Table 1:** Average power for electrodes and frequency bands during the discrimination period (power values are presented as Z scores and therefore may be negative. Values are expressed as M ± SD and the *p*-values were corrected using the Benjamini and Hochberg correction for false discovery rate). Values in bold indicate statistically significant differences.

	Delta			Theta			Alpha			Beta			Low Gamma		
Electrode	1st Block	2nd Block	*p* Value	1st Block	2nd Block	*p* Value	1st Block	2nd Block	*p* Value	1st Block	2nd Block	*p* Value	1st Block	2nd Block	*p* Value
Fp1	1.6611 ± 0.8673	1.4206 ± 0.6371	0.8641	−0.0086 ± 0.4489	−0.0339 ± 0.0978	0.8527	−0.0356 ± 0.1101	−0.0399 ± 0.0511	0.7127	−0.0445 ± 0.0207	−0.0449 ± 0.0258	0.4256	−0.0548 ± 0.0409	−0.0470 ± 0.0223	0.1069
Fp2	1.6826 ± 0.7694	1.7525 ± 0.8438	0.2072	−0.0124 ± 0.1211	0.0246 ± 0.1577	0.1363	−0.0408 ± 0.0831	−0.0361 ± 0.0748	0.4616	−0.0490 ± 0.0202	−0.0454 ± 0.0360	0.4835	−0.0558 ± 0.0317	−0.0601 ± 0.0287	0.7612
**F3**	2.9046 ± 0.6669	2.3935 ± 0.8941	0.6002	0.1372 ± 0.6375	0.2749 ± 0.2339	0.8163	0.0660 ± 0.1497	0.0495 ± 0.3234	0.1887	−0.0497 ± 0.0567	−0.0288 ± 0.0984	0.0795	**−0.0973 ± 0.0404**	**−0.0815 ± 0.0654**	**0.0160**
Fz	2.8447 ± 0.7748	2.8816 ± 1.0236	0.6997	0.4186 ± 0.5685	0.3041 ± 0.8422	0.3299	0.1063 ± 0.1268	0.0163 ± 0.2178	0.2558	−0.0709 ± 0.0386	−0.0554 ± 0.0361	0.2228	−0.1099 ± 0.0434	−0.1239 ± 0.0546	0.4455
F4	2.5040 ± 0.8625	2.4231 ± 0.7992	0.6014	0.0928 ± 0.3776	0.1634 ± 0.4769	0.3818	−0.0367 ± 0.1487	−0.0008 ± 0.1814	0.3192	−0.0559 ± 0.0242	−0.0554 ± 0.0282	0.4581	−0.0854 ± 0.0458	−0.0928 ± 0.0466	0.4481
T3	2.7204 ± 0.4576	1.9961 ± 0.6904	0.8002	0.2701 ± 0.3630	0.1503 ± 0.5923	0.3639	0.0617 ± 0.2182	0.0076 ± 0.1936	0.2710	−0.0234 ± 0.0672	−0.0409 ± 0.0987	0.2186	−0.0867 ± 0.0376	−0.0590 ± 0.0466	0.3314
**C3**	2.8788 ± 0.7331	2.1428 ± 0.8343	0.9253	0.2385 ± 0.6844	0.2480 ± 0.4386	0.8802	0.0844 ± 0.3681	0.2163 ± 0.2558	0.3507	−0.0516 ± 0.1169	−0.0450 ± 0.1522	0.2782	**−0.0983 ± 0.0422**	**−0.0618 ± 0.0828**	**0.0358**
Cz	2.2233 ± 0.6967	2.6410 ± 0.9653	0.2917	0.3027 ± 0.3109	0.1563 ± 0.4816	0.5320	0.0246 ± 0.1052	−0.0175 ± 0.0994	0.7712	−0.0688 ± 0.0264	−0.0667 ± 0.0301	0.6554	−0.1103 ± 0.0321	−0.0986 ± 0.0530	0.6685
C4	2.7756 ± 0.8746	2.8074 ± 0.8181	0.4940	0.1464 ± 0.6937	0.3296 ± 0.5377	0.4436	−0.0016 ± 0.2755	0.0660 ± 0.3921	0.1679	−0.0515 ± 0.0367	−0.0649 ± 0.1454	0.2535	−0.0908 ± 0.0526	−0.1081 ± 0.0896	0.4415
T4	2.2457 ± 0.8514	2.2777 ± 0.7393	0.6964	0.1027 ± 0.6003	0.1577 ± 0.7796	0.5702	0.0231 ± 0.3943	0.0756 ± 0.1252	0.7316	−0.0503 ± 0.2430	−0.0258 ± 0.0534	0.5429	−0.0839 ± 0.0379	−0.0677 ± 0.0462	0.1720
P3	3.0268 ± 0.5750	2.2269 ± 0.7362	0.9644	0.4237 ± 0.8182	0.3259 ± 0.5069	0.6983	0.0413 ± 0.2015	0.1310 ± 0.2593	0.1783	−0.0742 ± 0.0571	−0.0529 ± 0.0648	0.2880	−0.1300 ± 0.0382	−0.0956 ± 0.0572	0.0819
Pz	2.8522 ± 0.7742	2.2218 ± 0.7133	0.9450	0.5234 ± 0.4923	0.3734 ± 0.9479	0.3020	0.0017 ± 0.2819	0.0759 ± 0.2900	0.3005	−0.0799 ± 0.0412	−0.0694 ± 0.0236	0.5180	−0.1166 ± 0.0432	−0.1076 ± 0.0374	0.2758
P4	2.4519 ± 0.5919	2.1943 ± 0.6952	0.6558	0.4864 ± 0.5340	0.1935 ± 0.9309	0.3503	0.0162 ± 0.1712	0.0513 ± 0.3989	0.1097	−0.0685 ± 0.0339	−0.0647 ± 0.0384	0.2538	−0.1099 ± 0.0393	−0.0857 ± 0.0609	0.5894
O1	2.4528 ± 0.5793	2.8235 ± 0.7791	0.4849	0.7438 ± 0.5566	0.3474 ± 0.8173	0.4640	0.1496 ± 0.1841	0.2471 ± 0.2763	0.1267	−0.0070 ± 0.1817	0.0359 ± 0.2068	0.3725	−0.0971 ± 0.0480	−0.0746 ± 0.0631	0.4265
O2	2.4857 ± 0.8050	3.2250 ± 0.8105	0.3172	0.5660 ± 0.3712	0.4088 ± 0.6432	0.4056	0.1172 ± 0.2012	0.1062 ± 0.3738	0.2802	−0.0310 ± 0.0919	−0.0391 ± 0.1298	0.2722	−0.0761 ± 0.0399	−0.0871 ± 0.0426	0.5433
Tp10	2.3179 ± 0.8875	2.7886 ± 0.9398	0.4768	0.1308 ± 0.5248	0.1374 ± 0.9237	0.2401	−0.0138 ± 0.2505	0.0743 ± 0.2230	0.2909	−0.0403 ± 0.0456	−0.0378 ± 0.0508	0.3745	−0.0845 ± 0.0409	−0.0924 ± 0.0500	0.6949

**Table 2:** Rho values for latency and performance in electrode F4 (*p*-values corrected for multiple comparisons). The value in bold indicates a statistically significant correlation.

	Frequency Band	Rho	*p*-Value
Latency	Delta (0.3−4 Hz)	0.3562	0.24660
	Theta (4−8 HZ)	0.1419	0.57140
	Alpha (8−13 Hz)	0.0606	0.83100
	Beta (13−30 Hz)	−0.2719	0.38310
	Low Gamma (30−45 Hz)	−0.2246	0.38310
Performance	Delta (0.3−4 Hz)	−0.3453	0.24660
	Theta (4−8 HZ)	0.0435	0.83100
	Alpha (8−13 Hz)	0.2034	0.40270
	**Beta (13−30 Hz)**	**0.5536**	**0.020000**
	Low Gamma (30−45 Hz)	0.2554	0.38310
Latency × Performance	------------------------	−0.2364	0.38310

**Table 3:** Comparison of networks associated with performance in the present and the previous study.

	Passive width Discrimination Reference [10]	Active width Discrimination Present Study
Within-subjects (1st block->2nd block)	Parieto-occipital correlated to performance	Frontal correlated to performance

## Data Availability

The behavioral data, the neurophysiological data, and the code used for the present study are available at https://osf.io/qwbxg/.
